# Parent–child agreement on health-related quality of life (HRQOL): a longitudinal study

**DOI:** 10.1186/1477-7525-11-101

**Published:** 2013-06-20

**Authors:** Luis Rajmil, Amanda Rodríguez López, Sílvia López-Aguilà, Jordi Alonso

**Affiliations:** 1 IMIM, Institut Hospital del Mar d’Investigacions Mèdiques, Barcelona, Spain; 2 Agència d'informació, Avaluació i Qualitat en Salut (Agència de Qualitat i Avaluació Sanitàries de Catalunya), Barcelona, Spain; 3 CIBER en Epidemiología y Salud Pública, CIBERESP, Barcelona, Spain

**Keywords:** Adolescents, Health-related quality of life, Longitudinal studies, Parent–child agreement, Proxy

## Abstract

**Background:**

Few studies have evaluated changes on parent–child agreement in HRQOL over time. The objectives of the study were to assess parent–child agreement on child’s HRQOL in a 3-year longitudinal study, and to identify factors associated with possible disagreement.

**Methods:**

A sample of Spanish children/adolescents aged 8–18 years and their parents both completed the KIDSCREEN-27 questionnaire. Data on age, gender, family socioeconomic status (SES), and mental health (Strengths and Difficulties Questionnaire, SDQ) was also collected at baseline (2003), and again after 3 years (2006). Changes in family composition were collected at follow-up. Agreement was assessed through intraclass correlation coefficient (ICC), and Bland and Altman plots. Generalizing Estimating Equation (GEE) models were built to analyze factors associated with parent–child disagreement.

**Results:**

A total of 418 parent–child pairs were analyzed. At baseline the level of agreement on HRQOL was low to moderate and it was related to the level of HRQOL reported. Physical well-being at baseline showed the highest level of parent–child agreement (ICC=0.59; 0.53-0.65) while less “observable” dimensions presented lower levels of agreement, (i.e. Psychological well-being: ICC= 0.46; 0.38-0.53). Agreement parent–child was lower at follow-up. Some interactions were found between rater and child’s age; with increasing age, child scored lower than parents on Parents relationships and Autonomy (Beta [B] -0.47; -0.71 / -0.23) and the KIDSCREEN-10 (−0.49; -0.73 /-0.25).

**Conclusions:**

Parent–child agreement on child’s HRQOL is moderate to low and tends to diminish with children age. Measuring HRQOL of children/adolescents mainly in healthy population samples might require direct self-assessments.

## Background

Over the past years, a number of self-reported instruments assessing health-related quality of life (HRQOL) have been developed for children and adolescents. A systematic review identified almost a hundred instruments, 30 of them generic and more than sixty specific questionnaires addressed to this population by the year 2008 [1]. Most of the specific instruments collect information from proxies, mainly parents.

HRQOL assessment by proxies is controversial given the subjective nature of this concept. However, parents’ ratings are often used to assess HRQOL in young children, due to age, and/or lack of cognitive and linguistic skills necessary for self-completion of HRQOL measures [2]. Proxy-respondent bias represents a limitation on the assessment of HRQOL by parents [3,4]. Nevertheless, the parents’ perspective is frequently necessary given that they are responsible for the children and the ones who decide on their health needs and their use of healthcare services. This is an open research area where no consensus exists on when and how to collect and interpret information from parents on HRQOL [5].

Several factors have been described as associated to the degree and direction of parent–child agreement on the children’s HRQOL, mainly in cross-sectional studies. Parental well-being and child pain have been associated to disagreement in children with cerebral palsy [6]. Another study in children with chronic pain found no significant differences between self- and parents report on the PedsQL [7]. Direction of disagreement was also variable. A relatively high proportion of children with chronic conditions agreed with their parents while a low proportion of children scored lower (24%) and higher (32%) than their parents [8]. Some factors such as age [9], gender [10], parent’s age [11], or parental health [12-14] show variable, non-consistent results, while other factors such as child’s health status present more consistent results. In general, a moderate to high level of parent–child agreement was shown in children with chronic conditions [2,15,16], except for children with psychiatric conditions, such as depression, for whom a low level of agreement on their HRQOL was described [17]. Levels of agreement when comparing the evaluations of more objective dimensions, such as physical domains, are higher than those for more subjective aspects of health [9,11,14]. Direction of disagreement was also different according to the dimension of HRQOL analyzed [18].

Few studies have evaluated changes on parent–child agreement in HRQOL over time [19,20]. Some characteristics of these studies (such as small sample sizes or different instruments used for parents and children) call for additional longitudinal research. The magnitude and direction of this change can be clearly appreciated through longitudinal studies, since they allow identifying factors associated with changes over time and dimensions with marked changes in the degree of agreement.

The KIDSCREEN follow-up study [21] based on the Spanish sample of the European KIDSCREEN project [22], was designed to analyze changes on HRQOL over a period of 3 years of follow-up. We collected information of the children’s HRQOL both from the children themselves and from their parents at baseline and at a 3-year follow-up. The objectives of the present study were to assess parent–child agreement on HRQOL in a 3-year longitudinal study, and to analyze factors associated with these changes.

The main interest was to explore the association of age and time of follow-up on the level of parent–child agreement. According to the literature review, a higher level of agreement in those more “observable” dimensions was expected. It was also expected that the health status of both children and parents among other factors would be associated with the level of agreement at the follow-up.

## Methods

### Sample and data collection

This is a population-based longitudinal study. The target population for the KIDSCREEN study was children and adolescents aged 8–18. The aim was to recruit a sample that was representative by gender and 2 age groups (8–11 and 12–18 years old) in each participating country according to census data. Telephone sampling was centrally performed in Germany, and was carried out using a Computer Assisted Telephone Interview (CATI) with random-digital-dialling (RDD). Households were contacted by telephone and asked to participate by interviewers who had received study-specific training. If the family member contacted agreed to participate, the questionnaire and other study materials were mailed to their address together with a stamped, addressed envelope to return the completed questionnaire. A telephone hotline was used to provide further information about the survey. Two reminders were sent in cases of non-response (after two and five weeks) [23]. The Spanish KIDSCREEN baseline sample was recruited between May and November 2003 as part of the European KIDSCREEN fieldwork [22].

Between May and November 2006, follow-up questionnaires were mailed out to all children/adolescents and their parents who had agreed at baseline to participate in the follow-up study (840/926 participants). The fieldwork followed the same methodology as used at baseline [21]. Postal reminders were sent four and eight weeks after the first mailing to those who had not returned their completed questionnaires. A third reminder was sent after twenty weeks and any remaining non-respondents were contacted by phone.

All families participating in the study received a brief explanation together with separate questionnaires to be filled in by children and their parents independently. Participants were encouraged to maintain confidentiality between respondents. Moreover, at the end of the questionnaire some questions were included to collect information about difficulties and incidences during questionnaires completion.

All procedures were carried out following the data protection requirements of the European Parliament (Directive 95/46/EC of the European Parliament and of the Council of 24 October 1995 on the protection of individuals regarding the processing of personal data and on the free movement of such data). Signed informed consent was obtained from participants.

### The KIDSCREEN questionnaires

HRQOL was assessed through the KIDSCREEN-27 (27 items) [24,25] and the KIDSCREEN-10 [26]. KIDSCREEN items use 5-point answer categories to assess either the frequency (never-seldom-sometimes-often-always) or intensity (not at all-slightly-moderately-very-extremely). The recall period is 1 week. Scores for each dimension are calculated using Rasch analysis and then transformed into T-values with a mean of 50 and a standard deviation (SD) of 10. Higher scores indicate better HRQOL. The KIDSCREEN dimension scores refer to the mean values and standard deviation (SD) from a representative sample of the European general population. Separate questionnaires were administered simultaneously to the children and the parents to assess the HRQOL of the child. Each item of the parent version of the KIDSCREEN is reworded so it could be answered by a third person. For example, the question on the child/adolescent questionnaire “Have you been happy with the way you are?” was reworded on the parent questionnaire to, “Has your child been happy with the way he/she is?” To test our a priori hypotheses, the more ‘observable’ dimension of the KIDSCREEN-27 questionnaire (Physical well-being, 5 items) as well as the more ‘subjective’ dimensions (Psychological well-being, 7 items, and Parents relationships and Autonomy, 7 items) were included in the analyses.

### Other measures

Socio-demographic variables collected in the present study were age, sex, family socio-economic status, and parental level of education. Socio-economic status was measured using the Family Affluence Scale (FAS) [27], which includes family car ownership, having their own unshared room, the number of computers at home, and how many times the family spent on holidays in the past 12 months. FAS scores range from 0 to 7 and they were categorized as low (0–3), intermediate (4–5), and high (6–7) affluence level. Socio-demographic information collected from parents included the highest family level of education according to the International Standard Classification of Education (ISCED) categorized as low (at most lower secondary level, ISCED 0–2); medium (upper secondary level, ISCED 3–4); and high (university degree, ISCED 5–6) [28]. Baseline values for the FAS and Family level of education were used in the present analysis.

The Strengths and Difficulties Questionnaire (SDQ) is a brief behavioural screening questionnaire for children and adolescents aged 4–16 that asks about their mental health symptoms and positive attitudes [29]. The instrument consists of 25 items measuring 5 dimensions of emotional symptoms, conduct problems, hyperactivity/inattention, peer relationship problems, and pro-social behaviour. All items are scored on a three point scale with 0= not true, 1= somewhat true, and 2 = certainly true. Higher scores indicate more problems except on the pro-social behaviour dimension. Respondents were classified into 3 categories according to differences between 2003 and 2006 on the SDQ total difficulties score, as analyzed in a previous study [30]. The 3 categories were those who scored below −1 Standard Deviation (SD) from the mean (improved), those who scored above +1 SD (worsened), and the remainder of the respondents (stable).

Variables collected at baseline were age, sex and FAS from the child’s questionnaire, and family level of education, and parent perceived health from the parent questionnaire. The SDQ was collected at baseline and follow-up from the parent questionnaire.

#### Relevant events occurred between baseline and follow-up

Changes in family composition were collected at follow-up from the self-administered questionnaire and analyzed through a dichotomous variable (yes/no) from a list of possible changes between the baseline and follow-up assessments (i.e. parental divorce, or a death of a family member, or a birth of a new family member, etc.). Child’s self perceived health and parents’ own perceived health was elicited using a single question with 5 answer categories: “In general, how do you say your health is? Excelent, very good, good, fair, poor” (self-reported version). The number of missed school days and chronic conditions were collected both at baseline and follow-up from the parent questionnaire. The former was stratified into 3 categories: no days missed, 8 or more days in both contacts, and the rest of cases; and the latter was categorized as a dichotomous variable (yes/no) from a list of frequent conditions during childhood and adolescence.”

### Statistical analysis

Mean HRQOL scores were compared at baseline and follow-up using paired T Test. Agreement was analyzed through intraclass correlation coefficient (ICC) [31], Bland and Altman plots, and the 95% Confidence Interval (95%CI) was also calculated for the upper and lower limits of agreement [32]. The 95% CI of ICC was calculated to assess differences between baseline and follow-up administrations. An ICC lower than 0.4 was considered as very low, 0.4 to 0.74 as low to acceptable, and 0.75 or higher as excellent [33,34]. Analyses were carried out in the total sample as well as stratified by socio-demographic factors, health status and mental health.

Generalized estimating equation (GEE) models were built to identify factors associated with parent–child agreement. GEEs are an extension of generalized linear models to produce more efficient estimates than ordinary least squares regression in repeated measures studies because they account for the correlations between observations. Dependent variables were the KIDSCREEN-27 Physical and Psychological well-being, Parent relationships and Autonomy, and the KIDSCREEN-10 Index scores. The main independent variables included in the models were the assessment time (baseline 2003=0 and follow-up 2006=1), and the rater (1=child and 0=parent). Covariates included in the models were age, gender, the highest level of education of the family at baseline, changes in the family composition, chronic conditions and child’s mental health, and children and parents perceived health. Interaction terms between rater, age and assessment time were analyzed for a better identification of the change over time on the level of agreement. Bonferroni correction was used to address multiple comparisons.

Sample size was determined by initial participation rate, at the follow-up. The final sample size obtained allowed to detect a difference of 0.13 or higher in the ICC with an alpha error of 0.05, and beta =0.2.

## Results

The response rate at follow-up was 54% (n = 454). A total of 418 parent–child pairs with complete information were included in the analyses. Table 1 shows the sample characteristics of those included in the study; mean age at baseline was 12.6 (standard deviation, SD= 4.8); 55.5% of the sample was in the 10 to 13 years-old group at baseline, and the 49.3% reported intermediate FAS category. Mental health was stable in 75.4% of children, and perceived health at baseline and follow-up was very good for approximately 70% of the sample.

**Table 1:** **Characteristics of the participants in the Kidscreen follow**-**up study 2003**-**2006**

		**N (%)**	**N (%)**
		**Baseline (2003)**	**Follow-up (2006)**
**Sociodemographic characteristics**			
**Gender**	**Girls**	217 (51.9)	
	**Boys**	201 (48.1)	
**Age**			
	**Mean**, **SD**	12.6 (4.8)	
	**8** - **9 y**	68 (16.3)	-
	**10** - **13 y**	232 (55.5)	-
	**14** - **18 y**	118 (28.2)	-
**FAS**	**Low**	78 (18.7)	-
	**Middle**	206 (49.3)	-
	**High**	125 (29,9)	-
**Highest family level of education**	**Primary school**	154 (36,8)	-
	**Secondary school**	136 (32,5)	-
	**University negree**	110 (26,3)	-
**Changes in family composition**	**No**	-	353 (84,4)
	**Yes**	-	56 (13,4)
**Mental and physical health**			
**SDQ**	**Worsened**	-	39 (9,3)
	**Stable**	-	315 (75,4)
	**Improvement**	-	45 (10.8)
**Chronic conditions**			
**One or more**		125 (29.4)	
**Missed school days in the previous year**	**No days**	-	89 (21.3)
	**8 days or more**	-	19 (4.5)
	Some days missed*	-	310 (74.2)
**Children’s perceived health**			
**Self-reported**	**Good-fair-poor**	124 (29.9)	125 (30.1)
	**Excellent-very good**	291 (70.1)	290 (69.9)
**Parent’s characteristics**			
**Gender of respondent**	**Woman (mainly mothers)**	323 (77.3)	
**Age in 2003 (mean, SD)**		42.2 (4.8)	
**Parent self perceived health**			
	**Good-fair-poor**	96 (23.3)	110 (26.8)
	**Excellent-very good**	311 (76.7)	301 (73.2)

*FAS* Family Affluence Scale, *SDQ* Strengths and Difficulties Questionnaire, *HRQOL* Health-related quality of life. Missing values: age=1; *FAS*=9; Level of education=18; Family composition=5; *SQD*=19; chronic conditions=16; perceived health (baseline)=3, (follow-up)=3; parent self-perceived health (baseline)=12, (follow-up)=8. * Some school days missed in one of administrations.

Table 2 shows self- and parent-administered responses on children’s HRQOL both at baseline and follow-up as well as child- parent differences and 95% CI of the lower and upper limits of agreement. Statistically significant differences between self and parent reports were found on the dimensions of Physical well-being and Autonomy and relations with parents of the KIDSCREEN-27 at baseline (p<0.05). Mean child- parents differences ranged from 0.03 for Parent relationships and Autonomy at baseline to 1.76 on Physical well-being at baseline. Lower and upper limits of agreement showed great variability.

**Table 2:** Distribution of HRQOL scores according to the reporter

	**Self-reported**	**Parent-Reported**	**Paired T- test (p-values)**	**Mean diff. child – parent (SD)**	**95% confidence interval-lower agreement limit**		**95% confidence interval-upper agreement limit**	
	**Mean (SD)**	**Mean (SD)**						
**Kidscreen**-**27 Physical well**-**being (baseline)**	52.0 (11.0)	50.7 (9.9)	<0.001	1.76 (9.4)	−18.66	−15.44	18.96	22.16
**Kidscreen**-**27 Physical well**-**being (follow-up)**	48.5 (10.0)	47.9 (9.0)	0.17	0.69 (9.93)	−20.89	−18.05	18.83	22.27
**Kidscreen**-**27 Psychological well**-**being (baseline)**	53.2 (10.9)	53.5 (10.9)	0.53	−0.36 (11.41)	−25.14	−21.22	18.6	22.52
**Kidscreen**-**27 Psychological well**-**being (follow**-**up)**	50.6 (9.4)	51.6 (10.6)	0.06	−1.02 (11.11)	−25.12	−21.36	19.32	23.08
**Kidscreen**-**27 Autonomy and relations w/parents (baseline)**	53.1 (9.9)	54.6 (9.6)	0.01	−1.35 (10.54)	−24.27	−20.59	17.7	21.38
**Kidscreen**-**27 Autonomy and relations w/parents (follow**-**up)**	51.4 (8.9)	51.4 (8.2)	0.93	0.03 (9.38)	−20.35	−17.07	17.13	20.41
**Kidscreen**-**10 (baseline)**	53.6 (11.3)	53.8 (10.6)	0.94	0.04 (11.61)	−25.29	−21.15	23.26	25.33
**Kidscreen**-**10 (follow**-**up)**	49.8 (9.06)	50.7 (9.8)	0.11	−0.82 (10.26)	−23.12	−19.56	17.92	21.48

Kidscreen follow-up study 2003-2006.

**Figure 1:**
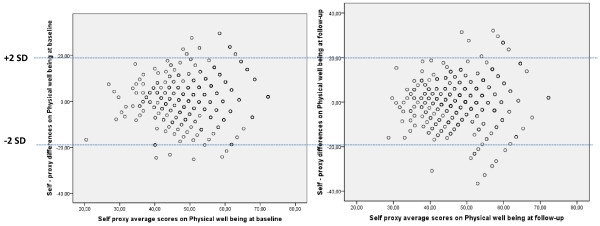
Bland & Altman plots of physical well-being (self and parent-reported Kidscreen-27) baseline and follow-up administrations.

**Figure 2:**
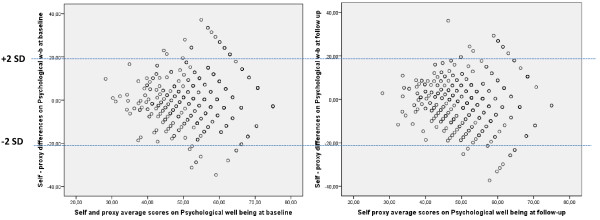
**Bland &Altman plots of psychological well-being (self and parent-reported Kidscreen-27) baseline and follow-up administrations.**

The level of agreement for Physical well-being varied according to the HRQOL level (Figure 1). Agreement was better for worst values of the Physical well-being, and slightly diminished at follow-up even in these worst values of HRQOL. Psychological well-being presented a similar distribution (Figure 2).

In general, ICC showed a level of agreement between low and moderate in both baseline and follow-up administrations. Approximately 20% of agreement was very low in both administrations (Table 3). Physical well-being showed the highest level of agreement at baseline (CCI=0.59; 0.53-0.65); ICC decreased in girls at the follow-up (ICC in 2003 for girls = 0.61; 0.52-0.69; and ICC in 2006 = 0.37; 0.24-0.48), and in the older age group (ICC in 2003 = 0.69; 0.58-0.77; and ICC in 2006= 0.42; 0.24-0.56), as the 95% CI did not overlap. The ICC for Psychological well-being was 0.46 (0.38-0.53) in 2003 and 0.39 (0.30-0.47) at follow-up (2006). No differences were found in the level of agreement neither in the total sample nor stratifying the sample in the dimensions of Parent relationships and Autonomy (baseline ICC = 0.42; follow-up ICC = 0.41) and the KIDSCREEN-10 (baseline ICC = 0.59; follow-up ICC= 0.44).

**Table 3:** **Parent**–**child agreement on children HRQOL** (**Physical and Psychological well**-**being**)

	**KD-27 Physical well**-**being (baseline)** **(2003)**	**KD-27 Physical well-being (follow-up) (2006)**	**Psychological well-being (baseline) (2003)**	**Psychological well-being – (follow-up) (2006)**
	**ICC (95% CI)**	**ICC (IC 95%)**	**ICC (95% CI)**	**ICC (95% CI)**
**HRQOL**				
**Total**	0.59 (0.53-0.65)	0.44 (0.36-0.52)	0.46 (0.38-0.53)	0.39 (0.30-0.47)
**Gender**				
**Girls**	0.61 (0.52-0.69)	0.37 (0.24-0.48)	0.43 (0.31 - 0.54)	0.34 (0.22 - 0.46)
**Boys**	0.55 (0.44-0.64)	0.48 (0.36-0.58)	0.48 (0.36 - 0.59)	0.43 (0.31 - 0.54)
**Age at follow**-**up**				
**11** - **12 y**	0.34 (0.10-0.54)	0.45 (0.23-0.62)	0.21 (−0.04 - 0.43)	0.37 (0.14 - 0.56)
**13** - **17 y**	0.52 (0.41-0.61)	0.39 (0.27-0.50)	0.43 (0.31 - 0.53)	0.26 (0.14 - 0.38)
**18** - **21 y**	0.69 (0.58-0.77)	0.42 (0.24-0.56)	0.51 (0.36 - 0.63)	0.56 (0.42 - 0.68)
**FAS**				
**Low**	0.60 (0.43-0.73)	0.34 (0.12-0.53)	0.43 (0.23 - 0.6)	0.41 (0.2 - 0.58)
**Middle**	0.64 (0.55-0.72)	0.46 (0.34-0.56)	0.51 (0.39 - 0.6)	0.43 (0.31 - 0.54)
**High**	0.50 (0.35-0.62)	0.43 (0.27-0.56)	0.36 (0.19 - 0.51)	0.29 (0.11 - 0.45)
**Parental level of education**				
**Primary school**	0.61 (0.50-0.70)	0.36 (0.20-0.50)	0.4 (0.26 - 0.53)	0.45 (0.31 - 0.57)
**Secondary school**	0.56 (0.43-0.67)	0.41 (0.25-0.54)	0.54 (0.4 - 0.65)	0.29 (0.13 - 0.44)
**University degree**	0.64 (0.52-0.74)	0.56 (0.41-0.67)	0.5 (0.34 - 0.63)	0.38 (0.2 - 0.53)
**Changes in family composition**				
**No**	0.60 (0.53-0.67)	0.41 (0.32-0.50)	0.47 (0.38 - 0.55)	0.38 (0.28 - 0.47)
**Yes**	0.52 (0.29-0.69)	0.64 (0.44-0.78)	0.4 (0.15 - 0.6)	0.49 (0.26 - 0.67)
**Health status of the Proxy**				
**Fair/poor**	0.55 (0.27-0.75)	0.04 (0.0-0.37)	0.53 (0.36 - 0.66)	0.35 (0.16 - 0.52)
**Good**	0.54 (0.43-0.64)	0.44 (0.31-0.55)	0.44 (0.31 - 0.55)	0.4 (0.26 - 0.51)
**Excelent/Very good**	0.63 (0.53-0.71)	0.48 (0.36-0.59)	0.26 (0.09 - 0.42)	0.32 (0.15 - 0.47)
**Missed school days**				
**No days (zero)**	0.56 (0.39-0.69)	0.40 (0.20-0.56)	0.49 (0.37-0.59)	0.35 (0.22-0.47)
**8 days or more**	0.37 (0.00-0.70)	0.05 (0.00-0.53)	0.55 (0.32-0.71)	0.57 (0.16-0.81)
Some days missed*	0.61 (0.53-0.68)	0.47 (0.38-0.56)	0.35 (0.21-0.48)	0.36 (0.10-0.58)

Kidscreen follow-up study 2003–2006.

*KD* kidscreen, *ICC* Intraclass correlation coefficient, 95 CI%: 95% confidence interval *Reporting missed some school days at baseline or follow-up.

Table 4 shows the results of GEE models. The main factors associated to parent–child disagreement were age, rater and perceived health. Interaction was found between age and rater: with each year of increasing child’s age, children scored lower than parents on Parent relationships and Autonomy (−0.47; -0.71 / -0.23), and on the KIDSCREEN-10 (−0.49; -0.73 /-0.25). An interaction was found between the rater and year of assessment: children scored higher than parents at follow-up (in 2006) on Parent relationships and Autonomy dimension (2.92; 1.38 /4.46).

**Table 4:** **Generalized estimating equation** (**GEE**) **models of factors associated to differences on parent**–**child responses over time**

	**Physical w-b**	**Psychological w-b**	**Parents and autonomy**	**KIDSCREEN**-**10**
	**Coefficient (95% CI)**	**Coefficient (95% CI)**	**Coefficient (95% CI)**	**Coefficient (95% CI)**
**Sex (male)**	3.13 (1.86 / 4.39)			
**Age**	−1.05 (−1.22 / -0.89)	−0.86 (−1.13 / -0.59)	−0.23 (−0.41 / 0.01)	−0.75 (−0.96 / -0.55)
**Rater (child)**	7.01 (3.24 / 10.77)		4.4 (1.27 / 7.7)	6.39 (2.95 / 9.82)
**Year of assessment (2006)**		−8.00 (−11.99 / 4.05)	−2.53 (−3.75 / -1.3)	−9.25 (−13.05 / -5.45)
**Child self**-**perceived health (exc**-**very good)**		4.02 (2.4 / 5.64)		
**Parent self**-**perceived health (exc**-**very good)**	5.52 (4.02 / 7.01)	2.58 (0.89 / 4.27)		
**Interaction terms**				
Rater X age			−0.47 (−0.71 / -0.23)	−0.49 (−0.73 / -0.25)
Rater X year of assessment			2.92 (1.38 / 4.46)	
Year of assessment X age		0.54 (0.27 / 0.81)		

Kidscreen follow-up study 2003–2006 (n=404-415).

Reference category: sex: female; rater: parent; year of assessment: 2003; level of education: university degree; child and parent self perceived health: good or less.

The level of statistical significance was corrected for multiple tests (Bonferroni correction = p<0.0015).

## Discussion

The present study is one of the few that analyze changes over time in the level of parent–child agreement on the kid’s HRQOL, based on a general population sample of children and adolescents. In general, the level of agreement was low to moderate in both the baseline and follow-up assessments, but tended to be lower at the follow-up. Child’s age and parent’s self-perceived health were the main factors associated to parent–child differences over time.

Few studies have analyzed parent–child agreement in a longitudinal design. One of these studies was carried out on 83 children with diabetes type I and analyzed HRQOL using the Child Health Questionnaire for parents (CHQ PF-50) and the self-reported version for adolescents (CHQ CF-80) from 12 to 18 years old [19]. Similarly, another study was carried out in a sample of 31 children with attention deficit hyperactivity, and HRQOL was evaluated through the Child Health and Illness Profile (CHIP-CE) which includes 44 items in the self-reported version and 75 items in the parent-reported version [20]. None of them carried out a formal comparison of longitudinal changes in the level of parent–child agreement. Our study included a sample of 418 parent–child pairs. The study allowed to evaluate changes over time in the level of agreement in a general population sample of children and adolescents, and to analyze factors with potential influence in these changes using an instrument with identical content for each respondent. Moreover, the HRQOL instrument used in the present study (KIDSCREEN) has demonstrated its ability to detect changes over time [35], which is a necessary psychometric property to determine the level of agreement between parent and child in a longitudinal design.

The results confirm those of previous studies that reported a higher level of agreement in the more observable dimensions [16]. Moreover, agreement on Physical well-being presented a decrement over time, and especially in girls and in the older age group. These results are consistent with one of the few longitudinal studies conducted in a population with diabetes type I in which agreement between adolescents and their parents also diminished over time in observable dimensions [19]. Other studies analyzing factors with potential influence on the level and direction of agreement showed a slightly higher level of parent–child agreement in children with cerebral palsy, compared with the results from the present study [6]. A general population study of parent–child agreement showed similar results to the present study, compared with our baseline results [18].

It is worth noting that the main factors associated to parent–child disagreement over time in the present study were age and to a lesser extent self-perceived health of both children and their parents. We found scarce or even no influence of other factors such as gender or chronic conditions in the level of agreement. The latter may be due to the healthy characteristics of the population sample analyzed in the present study. Different results could be found in children with serious chronic conditions and worse health because parents spend more time with the children helping with their care [6]. In this case, HRQOL could be assessed from both parent and child, or even just from the parent. This fact deserves attention for future studies.

The statistical method used to analyze agreement could be a factor with potential influence on the results. The most frequently used statistic for examining agreement between child and parent reports has been the Pearson product–moment correlation coefficient [2]. However, Pearson coefficients provide information on the covariation among scores but do not indicate absolute agreement [36]. A more appropriate statistic tool for examining agreement between raters is the ICC. ICC values provide an index that reflects the ratio between subject variability and total variability [37]. Moreover, the use of Bland and Altman method allowed graphical display of differences in the level of agreement and determine the degree of its variability [32]. For example lower and upper 95% CI for agreement on Physical well-being at baseline ranged from −20.89 to 22.27, a fact that discourages the use of parents as proxies on HRQOL assessment at individual level.

Some limitations of our study deserve comment. Response rates at follow-up (54%) could have biased the assessment of changes on HRQOL, and consequently, the level of agreement. Nevertheless, the response rate was similar to that in other longitudinal population-based studies [38], and although those followed up were slightly younger and from more educated families than non-participants, there were no significant differences in their baseline HRQOL scores [21]. Secondly, the sample size allowed us to detect a difference in the ICC of 0.13 (a relatively high difference). However, when we analyzed the agreement at the baseline contact including all parent–child pairs (n = 840), 95% CI were similar to those obtained at follow-up (data not shown). These data support the idea that the results are more related to variability in the level of agreement than to the small sample size. On the other hand, the present study included a higher sample size than other published studies [19,20]. Thirdly, some factors with potential influence on the level of agreement such as changes on some health-related behaviors (starting to smoke, drinking, etc.) and special needs were not included and should be taken into account in future studies. It is worth noting that response shift could have influenced the results on the level of agreement over time, although probabilities of this fact are low given that the sample characteristics and the time passed between baseline and follow-up could have made this less likely [39]. Finally, adolescents 19–21 years old would be expected to show a lower level of agreement than the rest of age groups. Nevertheless, very few differences were found in the results on this age group. This could be associated to the fact that most adolescents from the present study were still living with their families and very few of them declared to live independently from the nuclear family at follow-up.

### Implications

HRQOL measurement in children has improved in the last years. The results of the present study suggest that measuring HRQOL of children/adolescents requires direct self-assessments as much as possible, and especially in healthy population samples. Different approaches could be assessed in other contexts such as in children with chronic conditions. There may be more agreement in children with worse health and in these cases parent’s perspectives on HRQOL could add valuable information. Moreover, new and innovative approaches are needed to add to the knowledge in this area, such as simultaneous parent–child dyad assessment [40]. They also suggest that future studies should also assess the level of agreement using more than 2 measures over time, and analyzing the influence of individual, family and social factors on this agreement in larger samples.

## Competing interest

Authors declare that they have no conflicts of interest.

## Authors’ contributions

LR and JA designed the project and participated in data collection; AR, SLA and LR analyzed the data. All authors contributed to the data interpretation and writing the manuscript. All authors read and approved the final manuscript.
